# Properties of Exchange Coupled All-garnet Magneto-Optic Thin Film Multilayer Structures

**DOI:** 10.3390/ma8041976

**Published:** 2015-04-21

**Authors:** Mohammad Nur-E-Alam, Mikhail Vasiliev, Viacheslav A. Kotov, Dmitry Balabanov, Ilya Akimov, Kamal Alameh

**Affiliations:** 1 Electron Science Research Institute, Edith Cowan University, 270 Joondalup Drive, Joondalup, WA 6027, Australia; E-Mails: m.vasiliev@ecu.edu.au (M.V.); k.alameh@ecu.edu.au (K.A.); 2 Institute of Radio Engineering and Electronics, Russian Academy of Sciences, 11 Mohovaya St, Moscow 125009, Russia; E-Mail: kotov.slava@gmail.com; 3 Moscow Institute of Physics and Technology, 9 Institutskiy Per., Dolgoprudny, Moscow Region 141700, Russia; E-Mail: dima-mipt@mail.ru; 4 Experimentelle Physik 2, Technische Universität Dortmund, D-44221 Dortmund, Germany; E-Mail: ilja.akimov@tu-dortmund.de

**Keywords:** magneto-optic, garnet, multilayer thin films, magnetic media, nanophotonics, integrated optics

## Abstract

The effects of exchange coupling on magnetic switching properties of all-garnet multilayer thin film structures are investigated. All-garnet structures are fabricated by sandwiching a magneto-soft material of composition type Bi_1.8_Lu_1.2_Fe_3.6_Al_1.4_O_12_ or Bi_3_Fe_5_O_12_:Dy_2_O_3_ in between two magneto-hard garnet material layers of composition type Bi_2_Dy_1_Fe_4_Ga_1_O_12_ or Bi_2_Dy_1_Fe_4_Ga_1_O_12_:Bi_2_O_3_. The fabricated RF magnetron sputtered exchange-coupled all-garnet multilayers demonstrate a very attractive combination of magnetic properties, and are of interest for emerging applications in optical sensors and isolators, ultrafast nanophotonics and magneto-plasmonics. An unconventional type of magnetic hysteresis behavior not observed previously in magnetic garnet thin films is reported and discussed.

## 1. Introduction

Magneto-optic (MO) garnet materials, multilayer garnet-based thin film structures and nano-structured magnetic photonic crystals (MPCs) have attracted world-wide research interest recently due to their strong application potential in information technology and all-optical reconfigurable signal processing devices [1,2,3,4,5,6,7,8,9,10,11]. A number of research works have been conducted during the last two decades to design and fabricate magnetic multilayer thin film structures and photonic crystals incorporating ferrimagnetic, paramagnetic and dielectric materials of different composition types for use in applications ranging from the optical sensors to magnetic field visualizers and integrated optical isolators [4,5,6,7,8,9,10,11]. The effects of magnetic exchange coupling in magnetic thin films and multilayers (including interlayer exchange-coupled structures and magneto-elastically coupled systems) have also been attracting significant research attention due to the unique potential of achieving custom-engineered magnetic properties within these material system types [12,13,14,15,16,17,18].

It is always challenging to fabricate high-quality thin film nanostructures (of either the single- or multi-layer type) with a good degree of control over their microstructure, surface and interface quality, and the optical and magnetic behaviors, especially if the magnetic switching performance and magnetic anisotropy properties need to be adjustable. RF magnetron sputtering allows precise control over the thin film deposition process parameters, thus addressing the issues related to synthesizing high-quality thin films and multilayers wherein each layer’s chemical composition (stoichiometry) must be controlled accurately [10,11,19,20,21]. To investigate the potential of obtaining special magnetic properties that are not attainable easily using single-layer garnet thin films, we prepare all-garnet multilayer structures using two record-performance highly-Bi-substituted iron garnet materials having dissimilar magnetic behaviors (magnetic anisotropy, switching fields and saturation magnetizations). The bismuth-substituted iron garnet (Bi:IG) thin film materials pre-selected for constructing magnetic multilayers have previously been studied by our group in detail, and each possess extra-ordinary optical and MO property combinations. The reported performance characteristics of the four selected garnet material types of nominal stoichiometries Bi_2_Dy_1_Fe_4_Ga_1_O_12_, Bi_2_Dy_1_Fe_4_Ga_1_O_12_:Bi_2_O_3_, Bi_1.8_Lu_1.2_Fe_3.6_Al_1.4_O_12_, and Bi_3_Fe_5_O_12_:Dy_2_O_3_ have demonstrated that they not only possess exceptional optical and MO properties, but also attractive magnetic switching properties, especially when mixed with extra co-sputtered bismuth oxide during the RF sputtering deposition [19,20,21]. The present study is aimed at achieving controlled variations in the magnetic properties of all-garnet multilayers by making triple-layer structures composed of different high-performance magnetic material layers that (due to being in close proximity geometrically) interact with each other, leading to modified magnetic switching properties and demonstrating (in some cases) quite remarkable and unconventional magnetic switching behaviors. The material of composition type Bi_2_Dy_1_Fe_4_Ga_1_O_12_ possesses very high specific Faraday rotation (up to several thousand °/cm) in the visible range. This material possesses high uniaxial magnetic anisotropy (with its magnetization direction being perpendicular to the film’s plane) and also excellent magnetic memory properties (high remnant magnetization being in excess of 95% of its saturation magnetization). On the other hand, Bi-substituted lutetium iron garnet material of composition Bi_1.8_Lu_1.2_Fe_3.6_Al_1.4_O_12_ features a magneto-soft behavior with a significant in-plane magnetization component and also has high specific Faraday rotation [20].

In this paper, we report on the synthesis and properties of exchange-coupled all-garnet multilayer structures formed by sandwiching a layer of composition type Bi_1.8_Lu_1.2_Fe_3.6_Al_1.4_O_12_ or, alternatively, a nanocomposite described by the formula Bi_3_Fe_5_O_12_:Dy_2_O_3_, which have recently been developed by our group [21], in-between two layers of either Bi_2_Dy_1_Fe_4_Ga_1_O_12_ or a nanocomposite of type Bi_2_Dy_1_Fe_4_Ga_1_O_12_:Bi_2_O_3_. This synthesis approach is an extension of our previous work presented initially in [22], using some of the new MO materials developed recently. The goal of present work is to explore the engineering of magnetic properties in garnet multilayers, and especially to identify the ways of adjusting the coercive force and magnetic switching and anisotropy properties by varying the component layer stoichiometries. The principal material property-related results achieved in our high-performance magnetic multilayers are discussed in Section 2; the experimental processes for multilayer structure formation and characterization are detailed in Section 3.

## 2. Results and Discussion

### 2.1. Properties of Substrate/Bi_2_Dy_1_Fe_4_Ga_1_O_12_/Bi_1.8_Lu_1.2_Fe_3.6_Al_1.4_O_12_/Bi_2_Dy_1_Fe_4_Ga_1_O_12_ Thin Film Multilayers

The physical thicknesses of each layer within the multilayer thin film structures were measured during the deposition processes using a quartz microbalance sensor as well as an *in-situ* laser reflectometer system. Also, after the deposition, we re-confirmed the actual layer thicknesses of the structure using specialized thickness-fitting software (details of fitting procedures are described in [19]) and the optical transmission spectra of both the as-deposited and post-annealed structures. After depositing each batch of samples, several annealing trials were undertaken for each structure type, typically differing in the crystallization temperature by 5–15 °C, which often resulted in obtaining some “overannealed” samples with sub-optimal optical transparency properties. Transparency reduction was caused by film surface roughening appearing due to the formation of microscopic precipitates on film surfaces. The samples that had smaller-than–average specific Faraday rotations resulted typically from running the annealing processes at lower-than-optimum temperatures.

**Figure 1:**
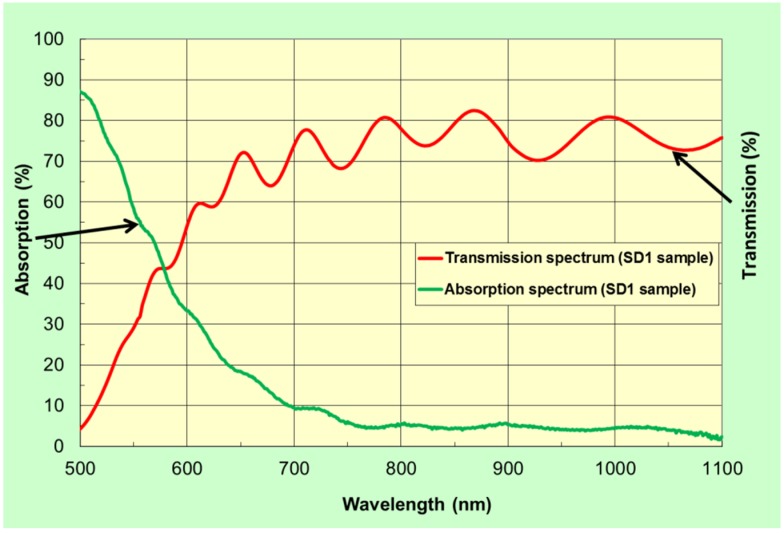
Transmission and absorption spectra of a multilayer garnet structure (SD 1); (red line) is the measured transmission spectrum of a structure, which was annealed with an optimized annealing regime (3 h at 630 °C) and (green line) is the measured absorption spectrum of this structure.

Figure 1 shows the transmission and absorption spectra of a three-layer garnet structure (Structural Design 1 (SD1) described in Table 1) obtained from an optimally annealed sample.

The measured transmission and absorption spectra of the annealed three-layer garnet structure were found very similar to the software-modeled transmission and absorption spectra with slight spectral transparency variations [22]. This is attributed to slight variations and non-uniformities in the refractive indices and (especially) due to the variations in the optical absorption spectra of both garnet types, which depend very strongly on the parameters of the thermal treatment regime. The measured reflection spectra of the developed samples were also performed using a UV/Visible spectrophotometer. A non-polarizing beam-splitter cube was used to adapt the transmission-mode spectrophotometer to make reflectivity measurements by reflecting a part of the light source beam off the cube’s diagonal plane and then off the samples, then off the diagonal plane again before propagating towards the detection system. A silver coating (200 nm silver layer deposited onto a glass substrate) was used as reference mirror placed in direct contact with the side of cube opposite to that against which the samples were placed. The reflection coefficients of the reference mirror and also of the cube’s diagonal were wavelength-dependent, but accurate calibration of the diagonal’s reflectivity was possible, and we used a spectrally-flat 97% reflectance approximation for our silver mirror. After system calibration, it was possible to calculate the sample’s own reflectance at each wavelength from the measured spectrophotometer transmission data, if transmission measurements were made sequentially with and without placing the sample next to the cube’s side surface. From the measurements of the transmission and reflection spectra, we derived the absorption spectrum of our three-layer garnet structure using the simple formula *A = 1-T-R* (%), where *A* is the absorbed power fraction, *T* is the power transmission coefficient, and *R* is power reflectivity. The variations in absorption data were also observed (compared to the modeled absorption spectrum) at wavelengths shorter than 700 nm can be attributed to the effects of scattering and also the differences in absorption coefficients between the thick single-layer materials and thin layers within multilayer structures [22].

**Figure 2:**
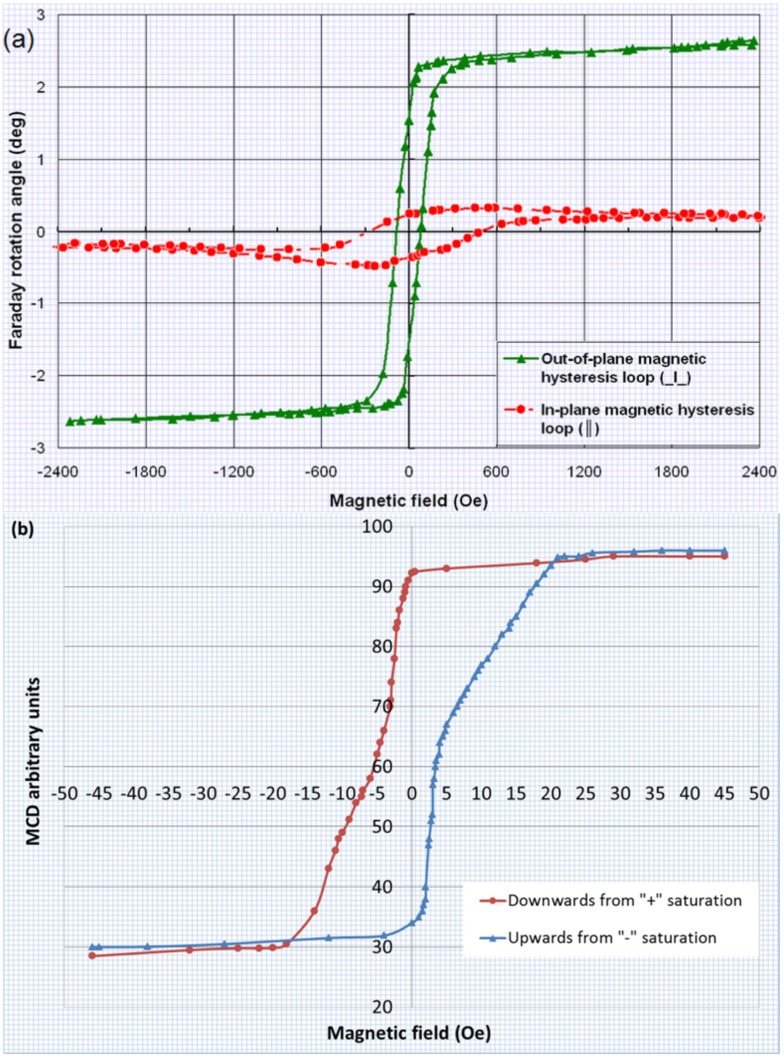
(**a**) Magnetic hysteresis loop obtained in all-garnet multilayer structure (SD 1) described as (Sub (GGG)/(500 nm Bi_2_Dy_1_Fe_4_Ga_1_O_12_)/(500 nm Bi_1.8_Lu_1.2_Fe_3.6_Al_1.4_O_12_)/(500 nm Bi_2_Dy_1_Fe_4_Ga_1_O_12_)) prepared on a GGG (111) substrate (and annealed for 3 h at 630 °C after the deposition) with an external magnetic field applied in the perpendicular (out-of-plane, green color curve) direction with respect to the film plane; (**b**) MCD hysteresis loop measured in an all-garnet multilayer structure (also of SD 1 design type) having thinner layer thicknesses; the graph has been digitized manually from the plotted MCD graph. The sample contained significantly thinner layers as described by (Sub (GGG)/(50 nm Bi_2_Dy_1_Fe_4_Ga_1_O_12_)/(50 nm Bi_1.8_Lu_1.2_Fe_3.6_Al_1.4_O_12_)/(50 nm Bi_2_Dy_1_Fe_4_Ga_1_O_12_)).

We performed magnetic hysteresis loop measurements for an annealed sample deposited onto a GGG substrate (from batch 1 of design type SD1) by applying an external magnetic field in both the perpendicular and also in-plane directions of the multilayer structure. The plane-polarized 532 nm laser beam used to polarimetrically measure the hysteresis loops of Faraday rotation in this sample (and all others) was always incident normally to the film surfaces, whilst the orientation of the substrates with respect to the applied magnetic field direction was varied when changing the measurement mode. Thus, the measured hysteresis loops traced the Faraday rotation angle changes occurring in response to the changes in the normal (out-of-plane) magnetization vector components in all layers of trilayer structures. The measured coercive force (for out-of-plane magnetization) was about 100 Oe, and the saturation field was around 200 Oe. In addition to rather low coercive force, a rather high uniaxial magnetic anisotropy (as evidenced by the large remnant magnetization) was also observed for this multilayer structure (the hysteresis loop measurement results are shown in Figure 2a). Figure 2b shows an out-of-plane magnetic hysteresis loop measured through magnetic circular dichroism (MCD) characterization in the structure of the same design type (SD1), but composed of much thinner garnet layers (each layer was only 50 nm thick).

We believe that within the garnet thin-film multilayer structures, exchange coupling between the different garnet layers of two different magnetic behavior types controlled the overall magnetic properties of these structures. The intermediate layer of garnet having an almost in-plane magnetization might have reduced the overall coercivity and switching field compared to that of the top and bottom magneto-hard garnet layers, whilst the strong perpendicular magnetization of these two outer layers helped inherit the magnetic memory properties in the switching behavior of the entire multilayer structure. The out-of-plane magnetic hysteresis loops feature a nearly-square shape and also high remanence, which is indicative of strong uniaxial magnetic anisotropy.

The comparison of two Faraday rotation hysteresis loops shown in Figure 2a reveals the opposite directions of the “saturation-level tilt”, which is explained, in the case of out-of-plane loop, by the effect of Faraday rotation still increasing at high fields in the 0.5 mm-thick paramagnetic (GGG) substrate. The data from the Faraday-effect hysteresis loop measured with in-plane-directed magnetic field does suggest the reduction in the perpendicular component of magnetization occurring in all layers with the increasing in-plane external field strength. The origins of very small coercive force observed in ultrathin trilayers (hysteresis loop data of Figure 2b) are related to the magnetization reversal mechanism which relies on the “seeding regions of magnetization reversal” and was shown to be expected in trilayer structures composed of similarly thin (≈50 nm) magnetic material layers [23].

### 2.2. Structures of Design Type SD2-Substrate/(Bi_2_Dy_1_Fe_4_Ga_1_O_12_: 17 Vol. % Bi_2_O_3_)/Bi_1.8_Lu_1.2_Fe_3.6_Al_1.4_O_12_/(Bi_2_Dy_1_Fe_4_Ga_1_O_12_: 17 Vol. % Bi_2_O_3_)

**Figure 3:**
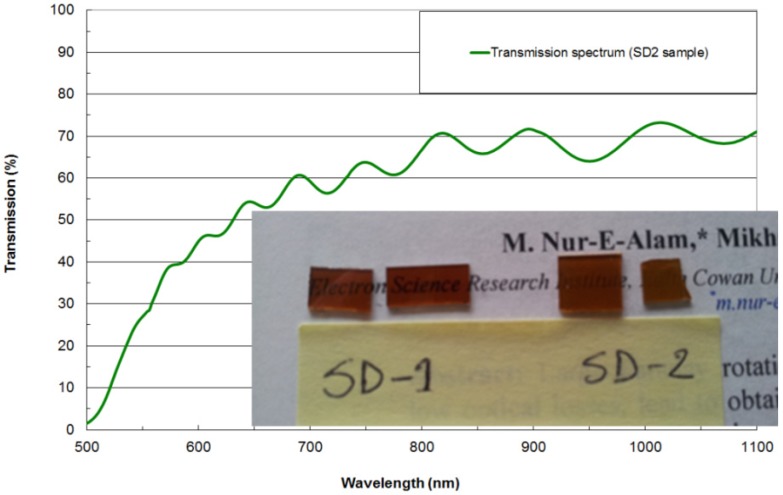
Measured transmission spectrum of a multilayer garnet structure (SD2) defined as (Sub (GGG)/(500 nm Bi_2_Dy_1_Fe_4_Ga_1_O_12_: 17 vol. % Bi_2_O_3_)/500 nm Bi_1.8_Lu_1.2_Fe_3.6_Al_1.4_O_12_/(500 nm Bi_2_Dy_1_Fe_4_Ga_1_O_12_: 17 vol. % Bi_2_O_3_)) prepared on a GGG (111) substrate and annealed for 1 h at 570 °C after the deposition. The inset shows a visual comparison of trilayer samples of two design types (SD1 and SD2).

In order to investigate the effects of incorporating nanocomposite garnet materials of type Bi_2_Dy_1_Fe_4_Ga_1_O_12_:Bi_2_O_3_ into trilayer exchange-coupled structures and simultaneously increase the Faraday rotation per unit structure thickness, two batches of trilayers of design type SD2 were deposited, onto the GGG and glass substrates, respectively. The amount of extra bismuth oxide content co-sputtered from a separate target (17 vol. %) was selected to facilitate a notable increase in specific Faraday rotation whilst at the same time avoiding a significant reduction in the upper limits of annealing temperature range (extensive data on these parameters and their inter-dependency has been reported in [19]). Additionally, according to the magnetic switching characterization results reported also in [19], changes in magnetic switching behavior compared to stoichiometric Bi_2_Dy_1_Fe_4_Ga_1_O_12_ layer behavior was expected due to the formation of nanocomposite oxide-diluted garnet-type material system. Structures of this design type were found to be rather difficult to anneal without losing some transparency (surface roughness features scattering light strongly across a broad range of wavelengths were forming during the crystallization processes, despite our efforts to find a processing regime optimized for the simultaneous crystallization of both material types). Figure 3 shows the measured transmission spectrum of an annealed SD2-type structure deposited onto a GGG substrate.

**Figure 4:**
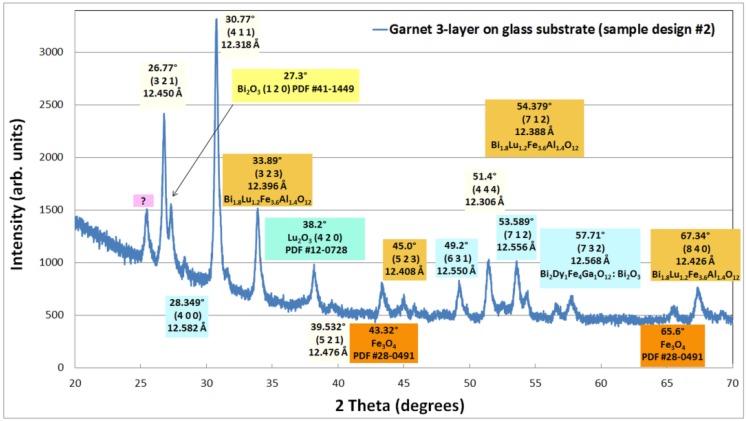
X-ray diffraction data set obtained from an annealed all-garnet multilayer sample of type SD2 (with individual layers of 500 nm thickness, deposited onto a glass substrate), which has demonstrated an unconventional magnetic hysteresis loop character. Several different types of crystallized garnet phases can be identified through their lattice constant values.

**Figure 5:**
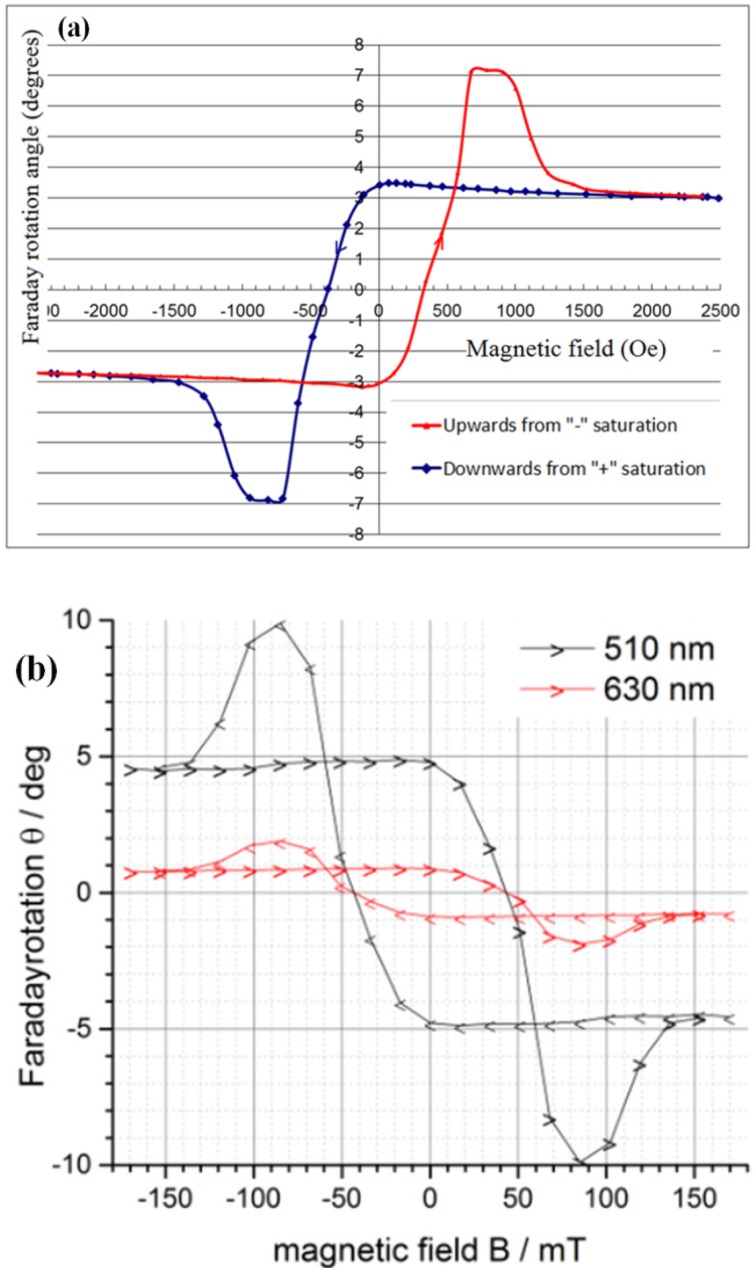

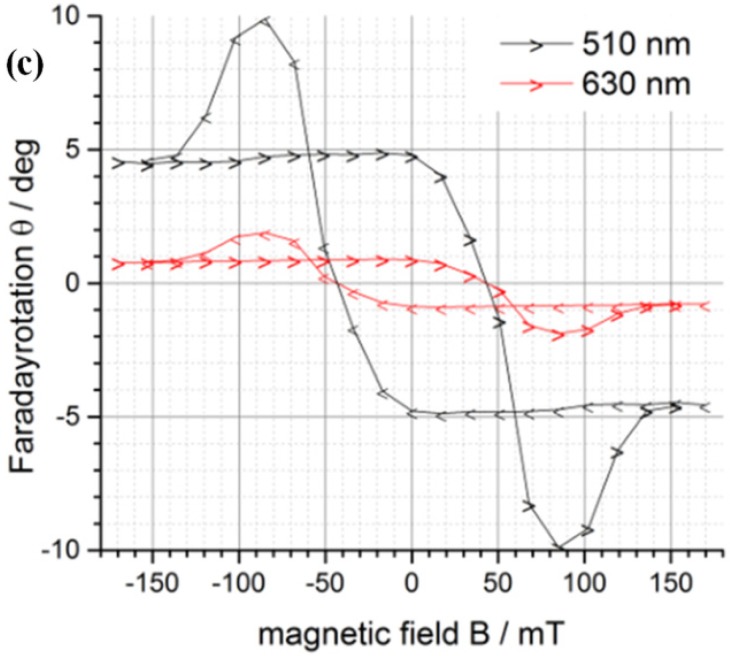
(**a**) An unconventional magnetic hysteresis loop measured in an all-garnet multilayer structure (SD2 with 500 nm individual layer thicknesses, deposited onto a GGG substrate) with an external magnetic field applied in the direction perpendicular to the film plane. Faraday rotation angle of the structure (for plane-polarized 532 nm light) was traced in transmission mode at different magnetization states using Thorlabs PAX polarimeter system. (**b**) Magnetic hysteresis loop of a multilayer structure (SD2 with 500 nm individual layer thicknesses, deposited onto a glass substrate), re-measured in another sample on glass substrate at different wavelengths. The external magnetic field was again applied in the direction perpendicular to the film plane. Faraday rotation angles of the structure (for plane-polarized 510 nm and 630 nm light beams incident normally) were traced in transmission mode at different magnetization states using a custom-made, splitting-cube-based polarimetry system. (**c**) Magnetic hysteresis loop of an SD2 multilayer measured using a custom-made polarimetry system in a sample deposited onto a GGG substrate.

After running the annealing crystallization processes and optical characterization, X-ray diffraction (XRD) measurements were carried out using detector-arm scanning technique. Near-grazing incidence of CuK_α1_ radiation onto film samples rotated slowly in the horizontal plane was used. The measurements were performed in the range of 2θ angles between 20° and 70°. The indexed X-ray diffraction data set obtained from a multilayer sample of design type SD2 deposited onto Corning Eagle XG glass substrate is shown in Figure 4. All samples from the same batch demonstrated a somewhat unconventional and unforeseen magnetic switching behavior type, illustrated in Figure 5. The angular positions of all X-ray diffraction peaks were determined by using the peak-listing options using Jade 9 (MDI Corp., Jakarta Pusat, Indonesia) software package. The data showed diffraction peaks at the sets of angles characteristic of the body-centered cubic lattice structure of garnets, and revealed their nanocrystalline microstructure. Several garnet-phase material types present within the structure were identified through indexing the diffraction lines shown in Figure 4, using their theory-predicted lattice constant values [24] and the standard lattice constant calculation methods described in [25]. The presence of several oxide material phases was also detected. In addition to confirming the crystallization of Bi_1.8_Lu_1.2_Fe_3.6_Al_1.4_O_12_ layer through identification of a cubic phase with its lattice parameter being near 12.4 Å (garnet material of this stoichiometry was expected to have a lattice parameter of 12.384 Å [20]), the crystallization of several garnet materials in the outer layers containing different Bi substitution levels were confirmed through indexing the XRD pattern data. The variations of lattice constants identified can be explained by the variations in the bismuth substitution content and indicate that some non-uniformity was present in the phase content and stoichiometry of the outer garnet layers.

A rather surprising type of magnetic switching behavior (illustrated in Figure 5) was observed during the Faraday rotation hysteresis loop measurements in all annealed samples of SD2 type, with the samples deposited onto both the glass and GGG substrates showing essentially the same switching behavior and similar Faraday rotations. A notable feature of hysteresis loop shape was the presence of an “intermediate saturation” state exhibiting the maximum Faraday rotation, which then reduced with external magnetic field increasing above about 1 kOe, reaching a saturation level near 1.6 kOe at below 50% of the maximum Faraday rotation angle reached at smaller fields. Since the trilayer structure does not feature any significant static (nor any field-strength-dependent) refractive index contrast between its layer materials, and neither is it a structure of any known photonic-crystal design type, the observed phenomenon of abrupt magnetic-field-dependent Faraday angle change is not caused by the optical interference-related effects. Also, no increased ellipticity was noted in the polarization state of the transmitted light, and thus the birefringence-related phenomena were not affecting the Faraday angle measurements. One possible explanation of this feature can be related to a magnetization reversal occurring within a part of the structure, e.g., the middle layer, in strong applied fields (caused by the exchange coupling phenomena of some unknown type), which thus could (in theory) lead to a change in the sign of specific Faraday rotation within the affected layer or volume. We have previously reported a theoretical analysis of magnetization dynamics and magnetization reversal processes in trilayers of the described type in [23], however no predictions were made in relation to the observed phenomenon. Furthermore, such magnetic behavior (reversal in the out-of-plane magnetization component direction) is completely unexpected to occur in increasing magnetic fields inside an already-saturated middle magneto-soft layer. We conclude that the most likely mechanism for this field-strength-dependent Faraday angle behavior is related to the effect of the large in-plane magnetization component within the middle layer (occurring in high external magnetic fields), on the reduction in the perpendicular magnetization components of outer nanocomposite layers. The remnant Faraday rotation angle difference between the out-of-plane and in-plane loops shown in Figure 2a was a factor of six (1.5° *versus* 0.25°), suggesting a six-fold reduction in the perpendicular magnetization component caused by the strong in-plane field. The in-plane magnetization component of the middle magneto-soft layer was also strong in high external fields, thus likely affecting the magnetic moments of garnet grains within the nanocomposite outer 500 nm-thick layers. The “negative-slope tilt” is again observed in the saturation behavior, similarly to the results shown in Figure 2a. The 4.2° (58%) reduction in the trilayer structure’s total Faraday rotation angle in high fields (peaking at 7.2° in low fields), if contributed by the two-thirds of the total optical path within structure, would correspond to a significant change in the perpendicular magnetization component in both outer layers (about 87% reduced perpendicular magnetization). However, the effect of very similar magnitude is also seen in Figure 2a, where a factor of six reduction in remnant Faraday rotation angle also corresponds to an 83% reduction in perpendicular magnetization component. The detailed mechanism of this phenomenon and its magnetization dynamics features will require further studies. This is certainly a phenomenon of notable interest for the development of specialized future types of magnetic storage media or integrated photonic devices for optical information processing.

The measurements shown in Figure 5a have been performed by the ECU (Australia) team, and the principal result in relation to the magnetic switching behavior has been confirmed independently by our Dortmund (Germany) co-author, whose measurement data are shown in Figure 5b,c. Since the principal magnetic switching-related data points (coercivity, the onsets and endpoints of the “intermediate saturation region” in all samples) have been measured to be independent of both the substrate type and the optical wavelength used to implement measurements, we conclude that the origins of the observed behavior are magnetics-related, rather than related to any optical interference or other optical effects. It is also important to note that the annealing crystallization regimes used to generate SD2 samples of Figure 5a–c have not been identical (570 °C for 1 h, 580 °C for 1 h, and 570 °C for 1 h, accordingly). Further studies of exchange-coupled magnetic multilayers containing different layer thicknesses, sequences and garnet material types will be necessary to fully explore the applications potential of this unconventional type of magnetic media.

### 2.3. Structures of Design Type SD3-Substrate/Bi_2_Dy_1_Fe_4_Ga_1_O_12_/(Bi_3_Fe_5_O_12_ + 6 vol. % Dy_2_O_3_)/Bi_2_Dy_1_Fe_4_Ga_1_O_12_

**Figure 6:**
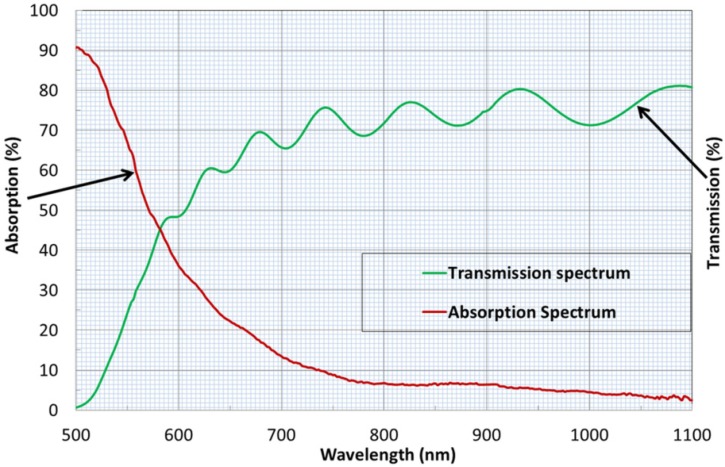
Measured transmission (green line) and absorption (red line) spectra of a multilayer garnet structure (SD3) described by (Sub (GGG)/(500 nm Bi_2_Dy_1_Fe_4_Ga_1_O_12_)/(500 nm Bi_3_Fe_5_O_12_: 6 vol. % Dy_2_O_3_)/(500 nm Bi_2_Dy_1_Fe_4_Ga_1_O_12_)) deposited onto GGG (111) substrate (and annealed for 3 h at 650 °C after the deposition).

**Figure 7:**
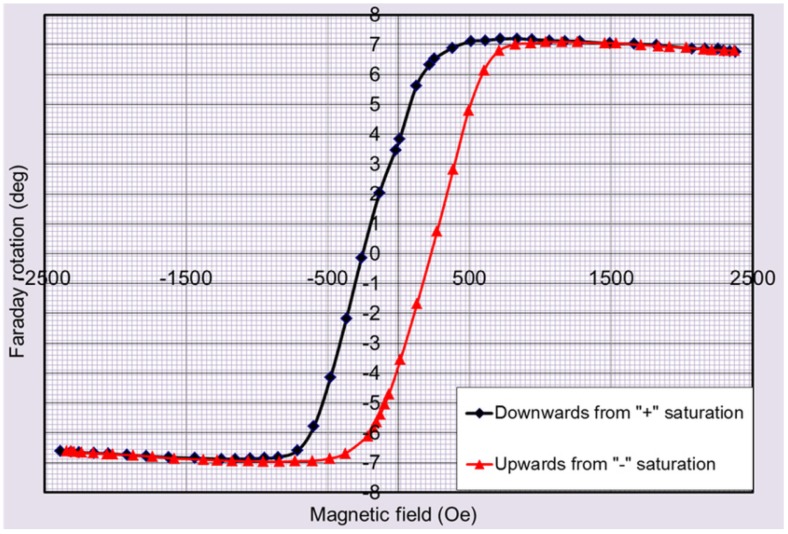
Magnetic hysteresis loop (measured by tracing the Faraday rotation angle at 532 nm at different magnetization states) of an all-garnet multilayer structure (SD3) with an external magnetic field applied in the direction perpendicular to the film plane of the structure.

Trilayer structures of design type SD3 were prepared to investigate the variations in the optical and magnetic properties of exchange-coupled trilayers induced by varying the middle layer composition. A 500 nm-thick composite thin-film material layer of type Bi_3_Fe_5_O_12_: Dy_2_O_3_ (6 vol. %) (a recently-developed type of high-performance magnetic garnets [21]) was placed in-between two Bi_2_Dy_1_Fe_4_Ga_1_O_12_ layers of the same thickness. An improved surface quality was seen in annealed trilayer samples compared to SD2 structures, and thus improved transparency was obtained (Figure 6 shows the measured transmission and absorption spectra). The saturated Faraday rotation measured in SD3 structures deposited onto GGG substrates exceeded that measured in SD1 trilayers by a factor of 2.7. At remnant magnetization, 2.5 times greater Faraday rotation angles were measured, when compared with structures of SD1 type. The magnetic properties of SD3 trilayers were characterized by measuring Faraday rotation hysteresis loop using 532 nm light (Figure 7). A combination of low coercive field value of about 240 Oe with relatively high Faraday rotation achieved in SD3 structures demonstrated that a strong potential exists for future multi-property optimization of highly bismuth-substituted exchange-coupled garnet trilayers.

The experimental results achieved demonstrate the possibility of engineering the magnetic properties of multilayer all-garnet structures using combinations of materials possessing different magnetic behavior types.

## 3. Synthesis and Characterization of Multilayer All-garnet Heterostructures

**Figure 8:**
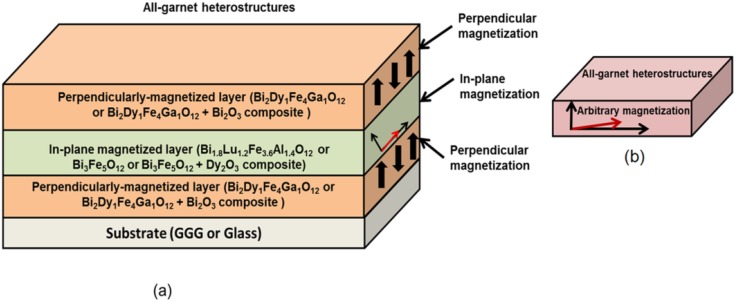
Schematic diagram of an exchange-coupled all-garnet trilayer structure (Substrate/Perpendicularly-magnetized garnet layer/In-plane magnetized garnet layer/Perpendicularly-magnetized garnet layer) indicating the types of MO garnet compositions and their individual magnetization behaviors (**a**) and the expected (hybrid-type) alteration of magnetic properties (magnetization vector direction) of the all-garnet multilayer structure (**b**).

Three batches of all-garnet multilayer structures of different layer materials combinations were prepared using the optimized deposition processes and an RF magnetron sputtering system. Highly bismuth-substituted iron garnet materials of composition types Bi_2_Dy_1_Fe_4_Ga_1_O_12_ (with perpendicular magnetization) and Bi_1.8_Lu_1.2_Fe_3.6_Al_1.4_O_12_ (with its magnetization vector being nearly in-plane) were used to fabricate the heterostructures. Figure 8 shows the schematic diagram of several structure designs including the material composition types and their magnetic behaviors. Inside the multilayer structures, a layer of magneto-soft, low-coercivity material (Bi_1.8_Lu_1.2_Fe_3.6_Al_1.4_O_12_ or Bi_3_Fe_5_O_12_:Dy_2_O_3_) was sandwiched in between two magneto-hard layers possessing strong uniaxial magnetic anisotropy (composition type Bi_2_Dy_1_Fe_4_Ga_1_O_12_ or Bi_2_Dy_1_Fe_4_Ga_1_O_12_:Bi_2_O_3_) of constant thickness, as shown in Figure 8a. Figure 8b shows the expected (and desired [22]) alteration of magnetic properties (magnetization vector direction) of the all-garnet multilayer structure.

A number of multilayer structures employing different garnet-type materials and also slightly different layer thicknesses were deposited onto gadolinium gallium garnet (GGG) and also onto glass (Corning Eagle XG) substrates using RF magnetron sputtering. The structures of each design type were prepared in a single deposition run by sequential sputtering of layers using oxide mix-based ceramic sputtering targets and low-pressure pure-argon plasma. The process parameters and conditions used to deposit these multilayers and also the optimized annealing regimes found to be suitable for the simultaneous crystallization of the structures (all three layers, each of which individually would have required a slightly different annealing process temperature and duration) to obtain very smooth layer interfaces, microstructure morphologies and microcrack-free surfaces are summarized in Table 1.

**Table 1:** Typical sputtering conditions and process parameters used to produce all-garnet multilayers.

Fabrication Process Parameters and Conditions/Sample Design	Sample Design 1 (SD1) 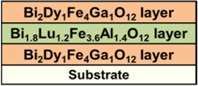	Sample Design 2 (SD2) 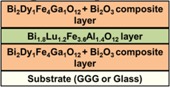	Sample Design 3 (SD3) 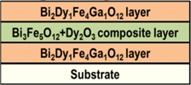
Sputtering targets (stoichiometries)	Bi_2_Dy_1_Fe_4_Ga_1_O_12_ and Bi_1.8_Lu_1.2_Fe_3.6_Al_1.4_O_12_	Bi_2_Dy_1_Fe_4_Ga_1_O_12_, Bi_1.8_Lu_1.2_Fe_3.6_Al_1.4_O_12_ and Bi_2_O_3_	Bi_2_Dy_1_Fe_4_Ga_1_O_12_, Bi_3_Fe_5_O_12_ and Dy_2_O_3_
Sputter gas and pressure	Argon (Ar), P(total) = 1 mTorr	Argon (Ar), P(total) = 2 mTorr	Argon (Ar), P(total) = 2 mTorr
Base pressure	P(base) < 1-2E-06 Torr (high vacuum)	P(base) < 1-2E-06 Torr (high vacuum)	P(base) < 1-2E-06 Torr (high vacuum)
RF power densities	6.09 W/cm^2^ for Bi_2_Dy_1_Fe_4_Ga_1_O_12_ target & 3.81 W/cm^2^ for Bi_1.8_Lu_1.2_Fe_3.6_Al_1.4_O_12_ target	6.09 W/cm^2^ for Bi_2_Dy_1_Fe_4_Ga_1_O_12_ target, 3.81 W/cm^2^ for Bi_1.8_Lu_1.2_Fe_3.6_Al_1.4_O_12_ target, & 0.55 W/cm^2^ for Bi_2_O_3_ target	3.67–4.78 W/cm^2^ for Bi_2_Dy_1_Fe_4_Ga_1_O_12_ target, 3.89 W/cm^2^ for Bi_3_Fe_5_O_12_ target & 0.99 W/cm^2^ for Dy_2_O_3_ target
Substrate surface temperature during deposition	250 °C	RT (20 °C)	RT (20 °C)
Substrate stage rotation rate	50–53 rpm	40–42 rpm	42–46 rpm
Deposition rate (measured by using a quartz microbalance sensor)	5–6 nm/min	4–5 nm/min	4–5 nm/min
Sample thickness (total) as deposited	150–1500 nm	1500 nm	1500 nm
Oven annealing regimes used (temperature and duration)	610–730 °C and between 1–10 h with 3 °C/min ramp-up and ramp-down rates	550–630 °C and between 1–6 h with 5 °C/min ramp-up and 10 °C/min ramp-down rates	590–660 °C and between 1–6 h with 3–5 °C/min ramp-up and ramp-down rates

High-temperature oven annealing processes (run using a conventional oven annealing system, CF 1200X, MTI Corporation) were applied to crystallize the as-deposited multilayer structures in order to synthesize the garnet phase in each layer. Numerous experiments were carried out to optimize the annealing regime for simultaneously crystallizing both material types within these garnet multilayer structures. A range of annealing temperatures between 550–730 °C and crystallization process durations ranging between 1–10 h were used. Besides the magnetic properties of these multilayers, we also characterized their optical and structural properties and compared some of these with the results of modeling. The transmission and reflection spectra of annealed multilayer structures were measured using a Beckman Coulter D 640B UV/Visible spectrophotometer. The measurements of total Faraday rotation angle of the structures and Faraday rotation hysteresis loops were performed using a Thorlabs PAX polarimeter system in conjunction with a custom-made calibrated electromagnet and a 532 nm plane-polarized laser source. X-ray diffractometry was used to reveal the crystal structure and material phase-related information as well as to identify the presence of different crystallized garnet phases within the same multilayer. X-ray diffraction (XRD) measurements were carried out using Siemens D5000 X-ray diffractometer.

## 4. Conclusions

Experimental studies of all-garnet multilayer structures incorporating two types of highly Bi-substituted iron garnet materials of high MO performance having dissimilar magnetic properties have demonstrated that customized magnetic properties tailored to specific application areas (e.g., integrated optics, photonics, and magneto-plasmonics) can be engineered within garnet multilayers. This has been our first in-depth experimental work on modifying the magnetic properties of garnet materials with the goal of reducing the coercive force whilst maintaining high specific Faraday rotation and remnant magnetization. Further studies will be conducted in order to prepare and characterize garnet multilayer structures having different combinations of high-performance garnet materials of various optimized thicknesses as well as different orientations of magnetization vectors within the component layers. These types of nano-engineered magnetic media are very important for multiple emerging applications in nano-photonics, especially for the design of optical sensors, magnetically switchable transparency elements and integrated optical isolators.
